# Inflammation of the Sinuses of the Face

**Published:** 1898-07

**Authors:** 


INFLAMMATION of the sinuses of the face are so frequent that they are found in 20 percent of the necropsies, and in 90 percent of those preceded by grip. V. Boland recommends remaining in bed if there is much reaction. Sudation with pilocarpin will sometimes afford much relief. He advises Moure's inhalations of the following: Add to a hot infusion of aromatic herbs a teaspoonful of the following: Pure alcohol, 150 grams; menthol, 5 grams; eucalyptol, 5 grams; oil of gaultheria, 5 drops; inhale for five minutes four times a day. Lermoyer recommends the following: Pure alcohol, 100 grams; menthol, 5 grams; add a tablespoonful to a bowl of hot water and inhale by the nose through a funnel for five minutes, every two hours. The Politzer douche cleanses the sinus in some cases. The contents of the sinus can also be aspirated out sometimes by deep inspirations, with the mouth and nose tightly closed.—*Gaz. Med. de Liege*, March 17.

